# Association between emotional distress and the efficacy of advanced gastric cancer patients undergoing treatment with immune checkpoint inhibitors: a cohort study and propensity score matching study

**DOI:** 10.3389/fonc.2025.1516643

**Published:** 2025-07-25

**Authors:** Ruoxi Tian, Siqi Wang, Zhengzheng Ji, Jiasong Li, Jingjing Zhang, Shasha Zhang, Zhanjun Guo

**Affiliations:** ^1^ Department of Colorectal Surgery, National Cancer Center, Beijing, China; ^2^ Department of Colorectal Surgery, National Clinical Research Center for Cancer, Beijing, China; ^3^ Cancer Hospital, Chinese Academy of Medical Sciences (CAMS), Beijing, China; ^4^ Department of Colorectal Surgery, Peking Union Medical College (PUMC), Beijing, China; ^5^ Department of Immunology and Rheumatology, The Fourth Hospital of Hebei Medical University, Shijiazhuang, Hebei, China

**Keywords:** gastric cancer, immune checkpoint inhibitors, emotional distress, depression, anxiety, prognosis

## Abstract

**Background:**

Cancer patients are at a greater risk of experiencing emotional distress (ED) compared to individuals without cancer, with those diagnosed with gastric cancer (GC) exhibiting a higher prevalence of ED than patients with other types of malignancies. A meta-analysis showed that 37% of global GC patients had depressive symptoms. Numerous studies have demonstrated that ED can lead cancer patients to develop immunosuppressive tumor microenvironments (TME), thereby impairing the exertion of antitumor immune effects. Currently, there is a lack of research investigating the correlation between ED and outcomes in GC patients undergoing treatment with immune checkpoint inhibitors (ICIs). We conducted a prospective cohort study to explore the correlation between ED and tumor response as well as prognostic outcomes in patients with advanced gastric cancer(AGC) who received ICIs treatment.

**Methods:**

We prospectively enrolled 104 patients with AGC undergoing combination therapy with ICIs, of whom 46 (44.2%) exhibited ED, defined as symptoms of depression (Patient Health Questionnaire-9 score ≥5) and/or anxiety (Generalized Anxiety Disorder 7-item scale score ≥5) at baseline. The Response Evaluation Criteria in Solid Tumors (RECIST v1.1) criteria were employed to evaluate tumor response. We analyzed the correlation between ED and outcomes including overall survival (OS), progression-free survival (PFS), objective response rate (ORR), and disease control rate (DCR).

**Results:**

Baseline ED was associated with a higher risk of death (HR: 2.035, 95%CI:1.272-3.254, P=0.003) and higher risk of progression (HR: 3.006, 95%CI: 1.922-4.701, P<0.001), as well as a lower DCR (RR: 0.504, 95%CI: 0.343-0.742,P=0.001), in AGC patients undergoing ICIs therapy. Cox multivariate analysis and propensity score matching (PSM) still indicated a significant correlation between ED status and survival outcomes. The baseline ED was not significantly correlated with cortisol levels with a HR of 2.318 (95% CI: 0.805-6.679, P=0.119). Patients exhibiting baseline depressive symptoms was correlated with reduced OS (HR: 2.231, 95%CI: 1.396 - 3.564, P=0.001) and PFS (HR: 2.488, 95%CI: 1.590 - 3.891, P<0.001) following ICIs therapy. After two cycles of treatment, the new onset of ED was found to have a worse survival prognosis compared to those who had never experienced ED (HR: 2.813, 95%CI: 1.270-6.228, P=0.011).

**Conclusions:**

ED is associated with worse outcomes in AGC patients undergoing treatment with ICIs.

## Introduction

The most recent report from the International Agency for Research on Cancer (IARC) indicates that in 2022, there were over 960,000 newly diagnosed cases of gastric cancer (GC) and nearly 660,000 deaths from the disease, making it the fifth leading cause of incidence and mortality globally (1). More than half of patients diagnosed with GC present with advanced-stage disease at the time of their initial diagnosis (2). For the treatment of advanced gastric cancer (AGC), the NCCN guidelines recommend a systemic therapy regimen based on platinum combined with fluoropyrimidine drugs, with immune checkpoint inhibitors (ICIs) or targeted therapy added to the regimen (3). However, as traditional chemotherapy drugs have reached a plateau, the choice of targeted drugs is limited, and the response rate of ICIs in some patients is suboptimal, leading to the poor overall prognosis of AGC with a 5-year survival rate of less than 10% (4). This disease not only poses a severe threat to human health but also imposes a heavy burden on healthcare systems due to enormous medical resource consumption, thereby exacerbating economic and social challenges for global development.

Immune checkpoints constitute a family of immunosuppressive molecules that interact with their corresponding ligands to attenuate the cytotoxic activity of T cells, thereby facilitating tumor immune evasion (5). ICIs can disrupt this immunosuppressive action by reactivating CD8+ T cells to secrete cytokines and exert cytotoxic effects for tumor cells elimination (6). The introduction of ICIs has significantly transformed the therapeutic landscape for patients with AGC. Before 2022, ICIs were predominantly recommended as second-line or third-line treatment options for AGC. In 2023, data from the phase III clinical trials CheckMate-649 (7), KEYNOTE-859 (8), and ORIENT-16 (9) revealed that the combination of ICIs with chemotherapy resulted in significantly improved clinical outcomes compared to chemotherapy alone. The results of the latest global phase 3 clinical trial (RATIONALE-305) suggested that the combination of tislelizumab and chemotherapy in the first-line treatment of AGC achieved a median overall survival (OS) of 17.2 months, with a 26% lower risk of death compared to chemotherapy alone (10). As a result, this combinatorial strategy has been formally acknowledged as the standard first-line therapy for AGC (3). Although ICIs have improved prognosis for GC patients, some patients have shown fewer responses to ICIs, with an objective response rate (ORR) of less than 20% (11). ICIs work by enhancing anti-tumor immunity within the tumor microenvironment (TME); the sensitivity and resistance to these agents are modulated by immune cells and cytokines (12). Prior research has indicated that the production of IL-6, IL-8, IFN-γ, CD8+ T lymphocytes, B cells, and tertiary lymphoid structures (TLS) are closely associated with responses to ICIs (13–16). Serum C-reactive protein (CRP), neutrophil-lymphocyte ratio, cortisol, growth hormone, and alpha-fetoprotein are also predictive factors for the efficacy of ICIs (15, 17–20). Identifying ICI efficiency associated for screening suitable populations can improve the prognosis of AGC patients.

Emotional distress (ED) is prevalent among cancer patients to such an extent that it is frequently designated as the sixth vital sign of cancer (21). ED, characterized by a disruption in psychological equilibrium, manifests in diverse forms. Common manifestations of ED include depressive and anxiety states, which can be assessed using standardized measurement scales (22). Depression predominantly presents as enduring feelings of sadness, diminished interest, and negative self-assessment, while anxiety is primarily characterized by restlessness, irritability, and heightened emotional sensitivity. Cancer-related symptoms, along with the psychological stress associated with diagnosis and treatment, significantly contribute to the occurrence of depression among cancer patients. Furthermore, inflammation induced by cancer itself may also play a mediating role in the onset of depressive disorders, resulting in an incidence rate of depression that is four times higher than that observed in the general population (23). A comprehensive large-sample study of cancer patients post-diagnosis revealed that 19.0% and 12.9% of these individuals demonstrate clinically significant levels of anxiety and depression, respectively (24). Notably, among the entire cohort of cancer patients, those diagnosed with GC exhibit a higher prevalence of comorbid anxiety and depression (25). A meta-analysis assessed the global prevalence of depression among GC patients, revealing that 37% of these individuals experience depressive symptoms (26). ED can reduce adherence, lower quality of life, increase the risk of suicide, and further increase the overall mortality rate in patients with GC. ED also affects the biological behavior of tumors at the molecular level. ED can impair the functioning of the central nervous system, exacerbate dysregulation of the hypothalamic-pituitary-adrenal (HPA) axis, and elevate the production of pro-inflammatory cytokines(IL-6, IL-8), potentially contributing to both the progression and severity of cancer (22, 23, 27–29).

ED, whose occurrence linked to immune-inflammatory mechanisms (30), may be associated with the efficacy of ICIs in cancer treatment. Previous study has reported that patients with melanoma who had ED prior to treatment had a higher risk of recurrence after surgery following neoadjuvant treatment with ICIs (31). A prospective observational study additionally found that ED was associated with poorer clinical outcomes in patients with non-small-cell lung cancer (NSCLC) undergoing ICIs as first-line therapy (22). Although GC patients have a higher incidence of ED, studies on the impact of ED on their outcomes with ICIs are scarce. Therefore, we conducted a prospective cohort study to investigate the association between baseline ED in AGC patients and tumor response as well as prognosis following ICIs therapy. Additionally, we investigated the effect of post-treatment changes in ED status on the prognosis of AGC patients undergoing ICIs therapy.

## Materials and methods

### Study design and participants

This study prospectively enrolled patients with AGC who were treated with ICIs for the first time at the Fourth Hospital of Hebei Medical University. The enrollment process was initiated on November 9, 2019, concluded on January 3, 2024, and follow-up activities were completed on June 30, 2024. The ICIs employed in this study are all anti- programmed cell death protein 1 (PD-1) drugs, which include pembrolizumab, toripalimab, camrelizumab, tislelizumab, and sintilimab. The targeted drugs used include tyrosine kinase inhibitors (Lenvatinib, Regorafenib) and the human epidermal growth factor receptor 2 antibody (trastuzumab). The chemotherapy drugs used include paclitaxel, platinum and fluoropyrimidine. The dosage and method of the drug were in accordance with the instructions of the drug. This study was approved by the Ethics Committee of the Fourth Hospital of Hebei Medical University, Written informed consent was signed by all patients.

The following criteria need to be met for inclusion: (1) Age ≧ 18 years old; (2) Patients diagnosed with GC through pathological examination; (3) According to the 8th American Joint Committee on Cancer (AJCC) staging system, patients with stage III or IV and no surgical indications; (4) Patients with at least one assessable target lesion; (5) Patients receiving anti-PD-1-based combination therapy; (6) Patients who have not received ICIs therapy before; (7) Patients who had chest and abdominal computed tomography (CT) scans within 1 week of starting ICIs therapy; (8) patients with 0–1 by Eastern Cooperative Oncology Group performance status (ECOG-PS) score; (9) Be informed and consent to participate in this study.

The following criteria need to be met for exclusion: (1) Patients with gastric stromal cell tumor or gastric neuroendocrine tumor; (2) Patients with a history of malignant tumors of other organs; (3) Patients with brain metastasis; (4) Patients currently receiving treatment for depression or anxiety. (5) Patients who cannot cooperate with psychological scale assessment.

### Patient outcomes

The evaluation of tumor response is conducted using CT scans after every 2 or 3 treatment cycles, following the guidelines outlined in version 1.1 of the Response Evaluation Criteria in Solid Tumors (RECIST v1.1) (32). The physicians performing imaging efficacy assessment and those responsible for administering ED questionnaires were kept mutually blinded, with no knowledge of each other’s work contents or information records.

Progression-free survival (PFS) was determined as the duration starting from the initial administration of ICIs therapy until disease progression, mortality, or conclusion of the study. OS was defined as the period commencing ICI-based systemic treatment and ending with death or study termination. ORR was determined by calculating the proportion of patients who exhibited a complete response (CR) or partial response (PR). Disease control rate (DCR) was calculated based on the percentage of patients with a CR, PR or stable disease (SD).

### Data collection

Depressive symptoms are evaluated using the 9-item Patient Health Questionnaire (PHQ-9), where a score of 5 or higher signifies the presence of depressive symptoms; anxiety symptoms are assessed through the 7-item Generalized Anxiety Disorder Questionnaire (GAD-7), with a score of 5 or higher indicating the existence of anxiety symptoms. When included patients completed the self-assessment questionnaire, a designated researcher was involved to assist them in minimizing confusion and ensuring accurate expression of their inner feelings. Patients exhibiting symptoms of depression or anxiety at baseline were classified as the ED group, whereas those without such symptoms were categorized as the no-ED group. The questionnaire survey was conducted within 1 week before ICIs treatment, and the anxiety and depression status scale was evaluated again after 2 cycles of treatment.

The PHQ-9 is extensively utilized for the evaluation of depressive symptoms among patients with cancer (33). This 9-item questionnaire encompasses the symptomatology of major depressive disorder as delineated by the American Psychiatric Association (34). Each item is rated on a scale from 0, indicating ‘not at all,’ to 3, representing ‘nearly every day,’ with an aggregate maximum score of 27 and a higher score indicates more severe depressive symptoms. In addition, the PHQ-9 has been translated into Chinese and validated in clinical research, and a threshold score of 5 demonstrates robust reliability and validity in the identification of depressive symptoms, exhibiting a sensitivity of 0.91 and a specificity of 0.77 (33, 35).

The GAD-7 is a validated self-assessment tool endorsed by the American Society for Clinical Oncology for the screening of anxiety symptoms among cancer patients (36). This scale consists of 7 items, each rated on a scale of 0-3, with a total score ranging from 0–21 and a higher score indicates more severe anxiety symptoms. GAD-7 was also translated into Chinese and has been extensively utilized in clinical research. It has demonstrated robust validity and reliability for screening anxiety symptoms among cancer patients, evidenced by a Cronbach’s α of 0.91 (36, 37).

Blood samples for adrenocorticotropic hormone (ACTH) and cortisol testing are collected by nursing staff between 6 am and 10 am, and it was determined by electrochemiluminescence method in the laboratory department of the Fourth Hospital of Hebei Medical University. The reference range for ACTH is established at 7.2-63.3 pg/mL, while the normative range for cortisol is defined as 133–537 nmol/L. Among all the patients, only one had an ACTH level above the normal upper limit. To investigate the relationship between ACTH and ED, we used a receiver operating characteristic (ROC) curve to determine the optimal cutoff value of ACTH as 25.09 pg/ml. Of the 59 patients with baseline cortisol data, only 3 had elevated cortisol levels. We used an ROC curve to determine the optimal cutoff value for cortisol at 193.95 nmol/L.

From the electronic case records, we extracted pertinent information regarding the patient, including age, gender, body Mass Index (BMI), programmed cell death-ligand 1 (PD-L1) expression combined positive score (CPS), status of human epidermal growth factor receptor 2 (HER2), tumor-node-metastasis (TNM) stage, treatment regimen, treatment lines, microsatellite instability (MSI) status, ACTH level, cortisol level, IL-6, ECOG PS, tumor response, progression status, the date of progression, survival status, the date of death. Part of the death time was obtained by telephone follow-up.

### Statistical analysis

Statistical analysis was conducted using IBM SPSS 26.0, StataSE 16, R (v. 4.4.1) software. The chi-square test or Fisher’s exact test was employed to compare enumeration data. Calculate the optimal cutoff value based on the ROC curve. Survival curves were generated using the Kaplan-Meier method and compared using the log-rank test. To further reduce survival time bias, we performed a landmark analysis. False Discovery Rate (FDR) was computed using the Benjamini–Hochberg method and a 10% FDR threshold was used. Multivariate survival analysis was performed employing a Cox proportional hazards model. Utilize R (v. 4.4.1) to generate a forest plot for the Cox proportional hazards model. In order to balance the differences in baseline characteristics between the two groups, propensity score matching (PSM) analysis was conducted. Different models were compared using maximum likelihood ratio to select covariates that needed to be matched. Using logistic regression, propensity scores were calculated with the ED group as the reference. The 1:1 nearest neighbor matching method was employed with a caliper value of 0.05. After matching, PSM parallelism hypothesis test was performed to examine whether the matching results were balanced. Kaplan-Meier method was used again on the balanced data to plot survival curves and conduct Log-rank test for comparison between curves. A significance level below 0.05 was deemed to indicate statistical significance.

## Results

### Patient characteristics

**Figure 1:**
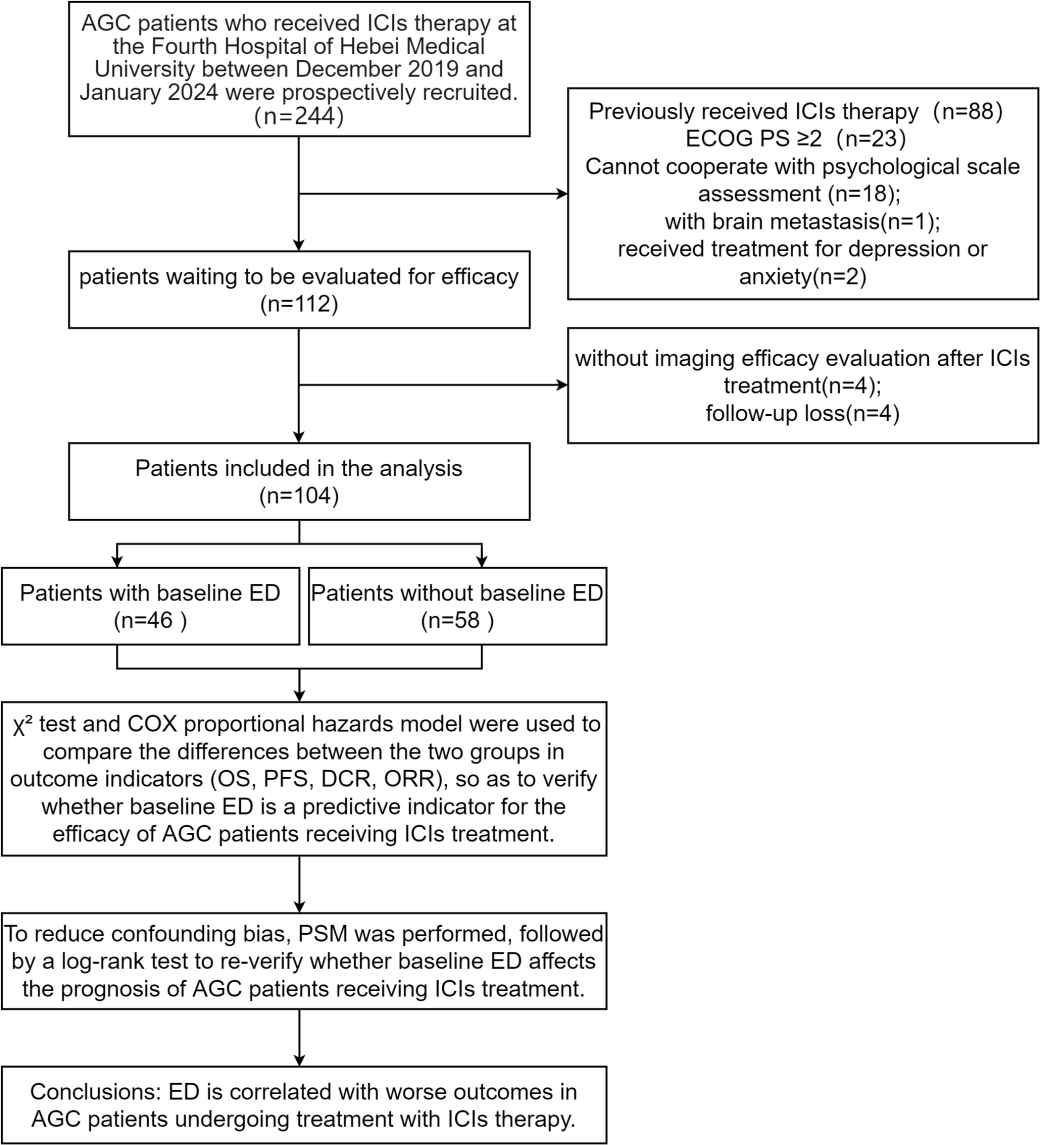
STROBE flow diagram.

**Figure 2:**
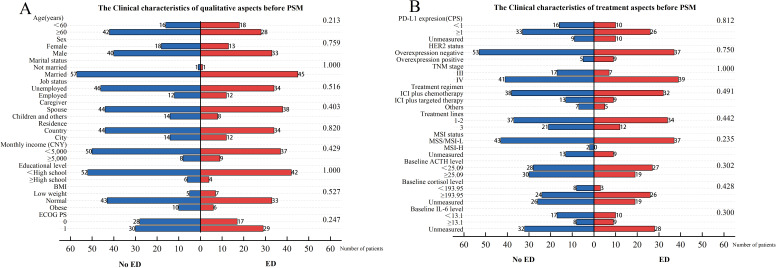
Clinical characteristics for included patients before PSM. The left vertical axis represents the covariates, while the right one corresponds to the P-values obtained from the chi-square test of each covariate against the groups of baseline ED. The horizontal axis reflects the number of patients. Among them, the blue bar graph represents the number of people in the baseline no-ED group, and the red bar graph represents the number of people in the baseline ED group. **(A)** The Clinical characteristics of qualitative aspects before PSM; **(B)** The Clinical characteristics of treatment aspects before PSM.

We initially enrolled 112 patients, excluded 4 due to lack of imaging assessment after ICIs therapy and lost 4 to follow-up, leaving 104 included in the data analysis. Flow diagram of the patient selection is presented in the STROBE flow diagram (**Figure 1**). Among the patients included, 46 (44.2%) were in the baseline ED group, while 58 (55.8%) were in the baseline no-ED group. Regarding treatment regimen, 70 (67.3%) patients were administered ICIs in conjunction with chemotherapy; 22 (21.2%) patients were treated with a combination of ICIs and targeted therapy, while 12 (11.5%) patients underwent a regimen that included ICIs, chemotherapy, and targeted therapy. No significant differences were observed in age, gender, marital status, job status, caregiver, residence, monthly income, educational level, BMI, PD-L1 expression CPS, status of HER2, TNM stage, treatment regimen, treatment lines, MSI status, ACTH level, cortisol level, ECOG PS, between the ED group and the no-ED group. The clinical feature data are summarized in **Figures 2A**, **B**.

The median follow-up time for the overall patients was 19.667 [95% confidence interval (CI): 15.637-23.697] months with 73 (70.1%) patients died during the follow-up period. The minimum follow-up time in this study was 55 days. The median OS for the entire patient cohort was 12.267 months (95%CI: 10.061-14.473), while the median PFS was 5.467 months (95%CI: 3.778-7.156). In terms of clinical efficacy assessment, none of the patients achieved CR, but PR was observed in 5 (4.8%) cases, and SD was seen in 58 (55.8%) cases, resulting in an ORR of 4.8%(95% CI: 0.6%-9.0%) and DCR of 60.6%(95%CI: 51.0%-70.1%) respectively (**Table 1**).

**Table 1:** Tumour response to ICIs therapy.

Response	Total No.	No ED	ED	RR	P
PD	41	13	28		
SD	58	40	18		
PR	5	5	0		
CR	0	0	0		
ORR	4.8% (0.6%-9.0%)	8.6% (1.2%-16.1%)	0.00%	0.114 (0.006-2.012)	0.138
DCR	60.6% (51.0%-70.1%)	77.6% (66.5%-88.6%)	39.1% (24.5%-53.8%)	0.504 (0.343-0.742)	0.001

### Evaluation of response to treatment

The ORR in the baseline no-ED group was 8.6% (95%CI: 1.2%-16.1%), while no patients had a tumor response of PR or CR in the baseline ED group, leading to no statistically significant difference between the two groups (P=0.138). The DCR was significantly different with 39.1%(95%CI: 24.5%-53.8%) for the ED group and 77.6%(95%CI: 66.5%-88.6%) for the no-ED group (RR: 0.504, 95%CI: 0.343-0.742, P=0.001, **Table 1**), these suggested that the no-ED group had a better response to ICIs.

### Evaluation of survival outcomes

**Figure 3:**
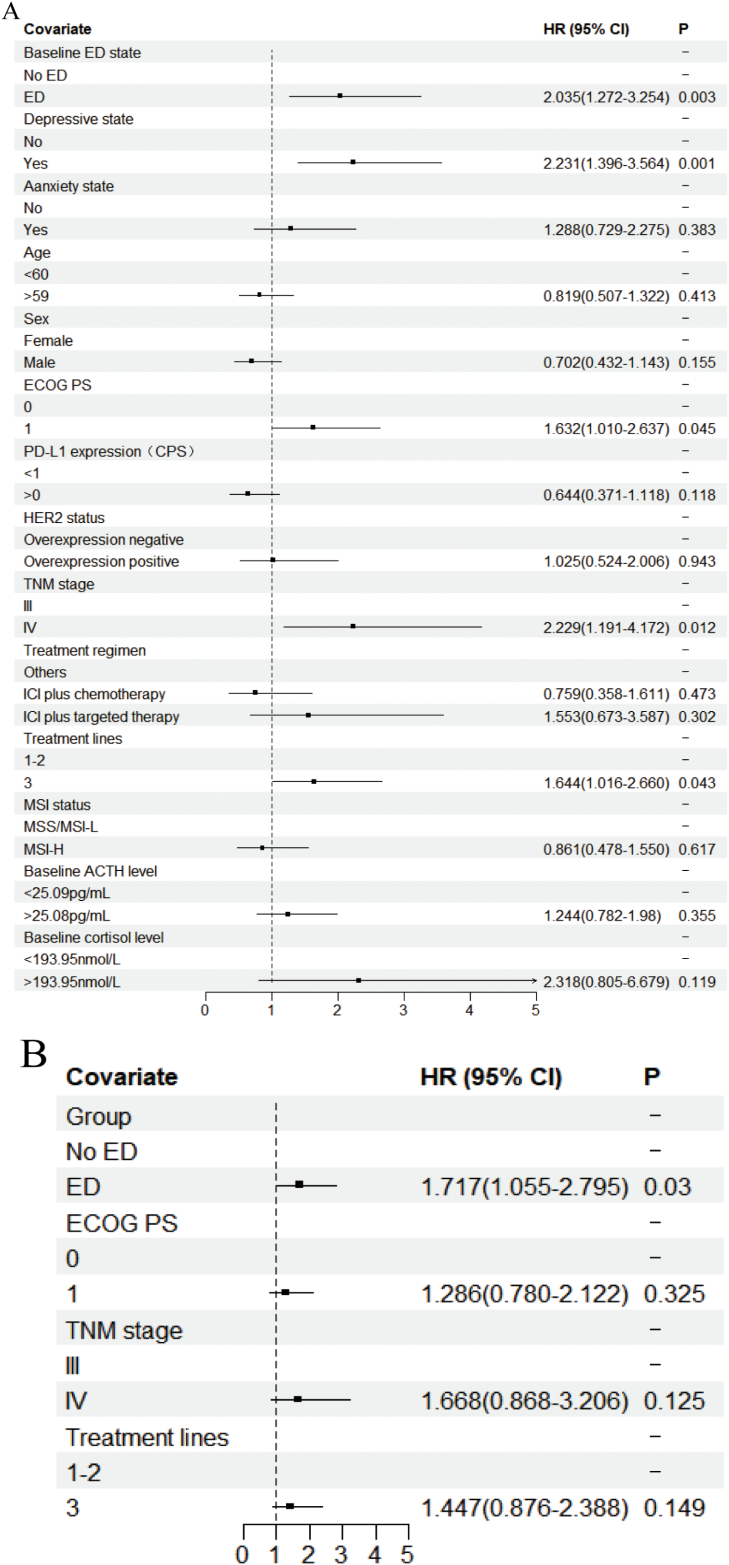
Forest plot of the Cox proportional hazards model in OS. Squares indicated study-specific HRs. 95% CIs are depicted by horizontal lines. The P-value is derived from the COX proportional hazards model. **(A)** Univariate analyse; **(B)** Multivariate analyse.

**Figure 4:**
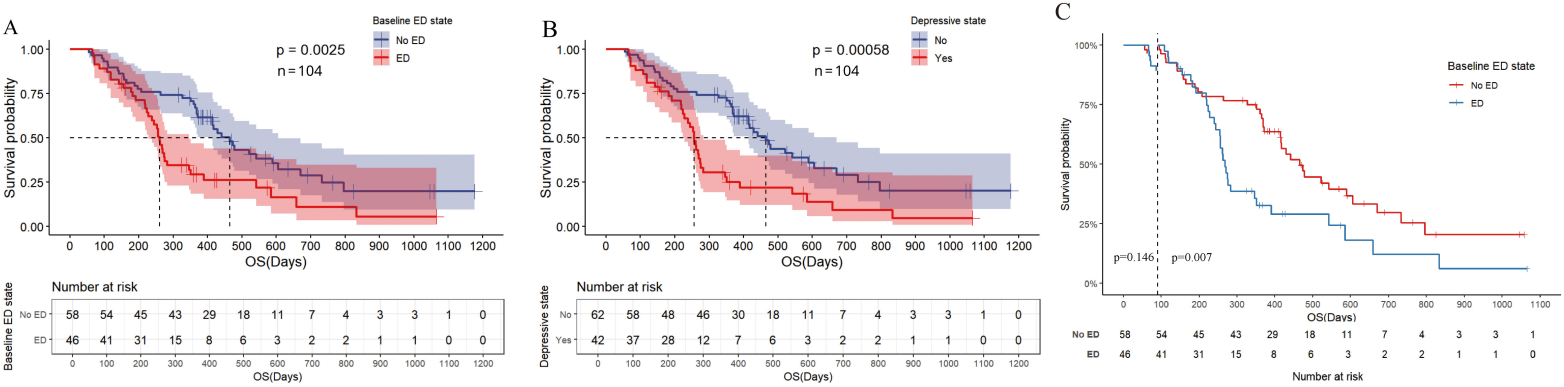
Kaplan Meier curve for OS before PSM. **(A)** OS of baseline ED state; **(B)** OS for depressive state; **(C)** Landmark analysis of OS according to baseline ED state.

In the univariate analysis of OS (**Figure 3A**, **Supplementary Table S1**), the median OS for patients in the baseline ED group was 8.700 months (95% CI: 8.102-9.298), compared to 15.500 months (95% CI: 13.423-17.577) in the baseline no-ED group. This difference was statistically significant with a hazard ratio (HR) of 2.035 (95% CI: 1.272-3.254, P=0.003), and remained significant after Benjamini–Hochberg correction. This suggested that patients in the baseline ED group had approximately double the mortality risk compared to those in the baseline no-ED group. For the p-values in the subgroup analysis of OS according to baseline ED state, the FDR was 7.1% (1/14), suggesting that baseline ED remained significantly associated with the risk of death in AGC patients receiving ICIs treatment after Benjamini–Hochberg correction (**Supplementary Figure S1**, **Supplementary Table S2**). The median OS for patients exhibiting a baseline depressive state was 8.533 months (95%CI: 7.801-9.265), whereas that for those without depressive symptoms at baseline was 15.500 months (95% CI: 13.158-17.842). This difference was statistically significant with a HR of 2.231 (95% CI: 1.396-3.564, P=0.001), indicating that a depressive state was significantly associated with poorer survival outcomes in AGC patients undergoing ICIs. The median OS for patients exhibiting a baseline anxiety state was 9.433 months (95%CI: 5.742-13.124), whereas the median OS for those without anxiety was 13.800 months (95%CI: 11.639-15.961). Although the median OS in the baseline anxiety group was shorter than that of the non-anxiety group, this difference did not reach statistical significance (HR: 1.288, 95% CI:0.729-2.275, P=0.383). In addition to the aforementioned factors, the ECOG PS (HR: 1.632, 95% CI: 1.01-2.637, P=0.045), TNM stage (HR: 2.229, 95% CI: 1.191-4.172, P=0.012), and treatment lines (HR: 1.644, 95% CI: 1.016-2.66, P=0.043) were found to be significantly associated with survival outcomes in AGC patients undergoing treatment with ICIs. Following a multivariate analysis incorporating 4 factors (**Figure 3B**), the mortality risk for the ED group remained significantly elevated compared to that of the no-ED group (HR: 1.717, 95% CI: 1.055-2.795, P=0.03), suggesting that ED independently correlated with adverse survival outcomes in AGC patients undergoing ICIs treatment. The OS-related Kaplan-Meier survival curves for baseline ED state was showed in **Figure 4A** and depressive state was showed in **Figure 4B**.

In the landmark analysis for OS according to baseline ED state, the risk of death in the baseline ED group was significantly higher than that in the no-ED group in the analysis after 3 months (P=0.007), while no significant difference was observed between the two groups in the analysis within 3 months (P=0.146), as shown in **Figure 4C**. Due to the median OS of AGC patients receiving ICIs combination therapy exceeding 1 year, and patients who did not undergo the second imaging evaluation not being included in the statistical analysis of this study, the proportion of deaths in both groups within 3 months was small (10.9% in the ED group and 6.9% in the no-ED group), which in turn caused the P-value of the log-rank test for OS within 3 months to fail to show a significant difference.

**Figure 5:**
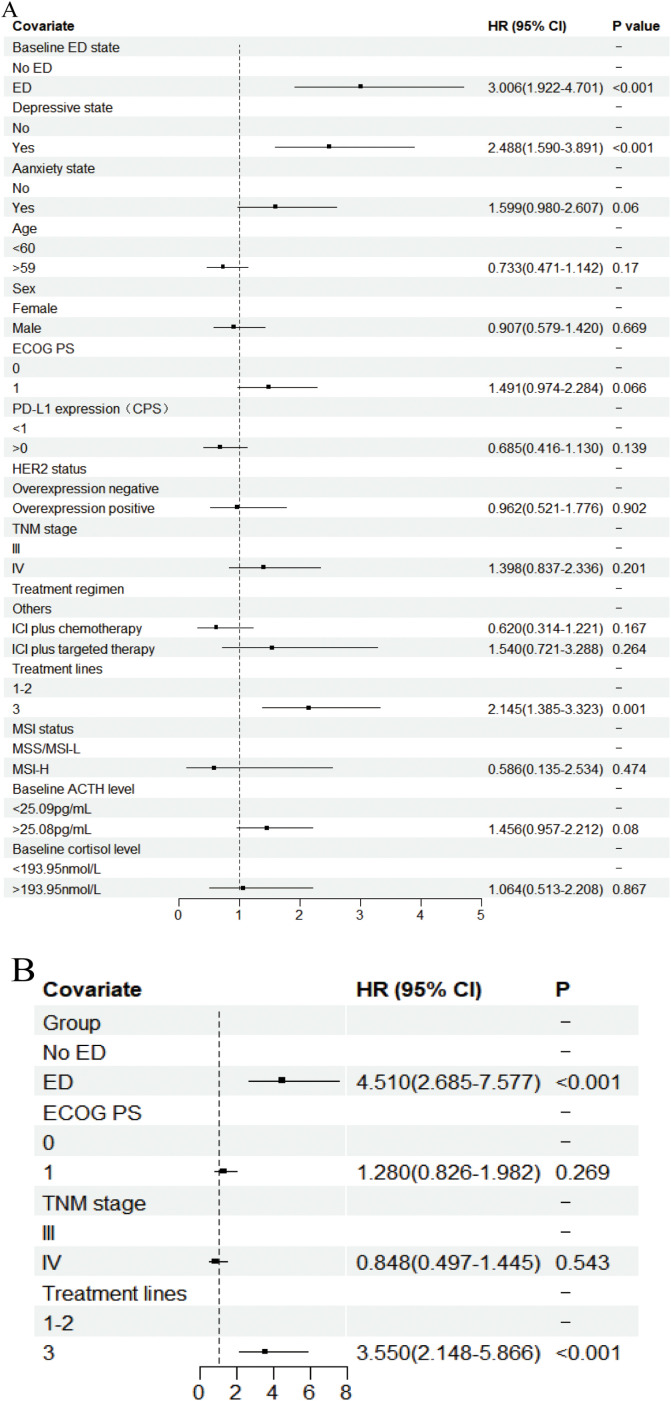
Forest plot of the Cox proportional hazards model in PFS. Squares indicated study-specific HRs. 95% CIs are depicted by horizontal lines. The P-value is derived from the COX proportional hazards model. **(A)** Univariate analyse; **(B)** Multivariate analyse.

**Figure 6:**
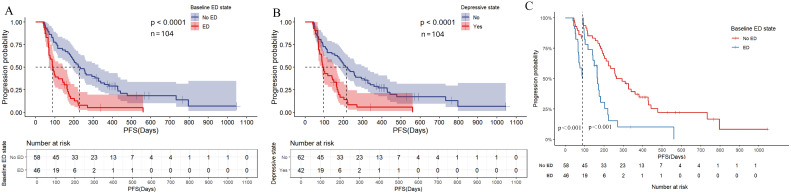
Kaplan Meier curve for PFS before PSM. **(A)** PFS of baseline ED state; **(B)** PFS for depressive state; **(C)** Landmark analysis of PFS according to baseline ED state.

In the univariate analysis of PFS (**Figure 5A**, **Supplementary Table S3**), patients in the ED group had a median PFS of 2.933 months (95% CI: 2.528-3.338), while the median PFS for patients in the no-ED group was 7.467 months (95% CI: 5.228-9.706). This difference was statistically significant with a HR of 3.006 (95% CI: 1.922-4.100, P < 0.001), indicating the risk of progression for patients experiencing baseline ED state was approximately threefold that of their counterparts without ED. For the p-values in the subgroup analysis of PFS according to baseline ED state, the FDR was 0 (0/21), indicating that baseline ED was still significantly associated with the risk of progression in AGC patients receiving ICIs treatment after Benjamini–Hochberg correction(**Supplementary Figure S2**, **Supplementary Table S4**). The median PFS for patients exhibiting a baseline depressive state was 3.100 months (95% CI: 2.571-3.629), whereas the median PFS for those without depressive symptoms was 7.033 months (95% CI: 4.911-9.155). This difference was statistically significant with a HR of 2.488 (95% CI: 1.59-3.891, P < 0.001), indicating that depressive state is associated with a higher risk of progression in AGC patients receiving ICIs therapy. Patients with baseline anxiety had a median PFS of 3.933 months (95% CI: 0.228 - 7.638), while those without anxiety had a median PFS of 5.833 months (4.157 - 7.509). The median PFS for patients with baseline anxiety state was shorter than that for non-anxious patients, but there was no statistically significant difference (HR: 1.599, 95% CI: 0.98-2.607, P=0.06). The results of multivariate analysis suggest that baseline ED is independently associated with the risk of progression in AGC patients received ICIs therapy (**Figure 5B**). The PFS-related Kaplan-Meier survival curves for baseline ED state was showed in **Figure 6A** and depressive state was showed in **Figure 6B**. In the landmark analysis for PFS according to baseline ED state, the progression risk in the baseline ED group was significantly higher than that in the no-ED group both within 3 months (P < 0.001) and after 3 months (P < 0.001), as shown in **Figure 6C**.

### Survival analysis after PSM

**Figure 7:**
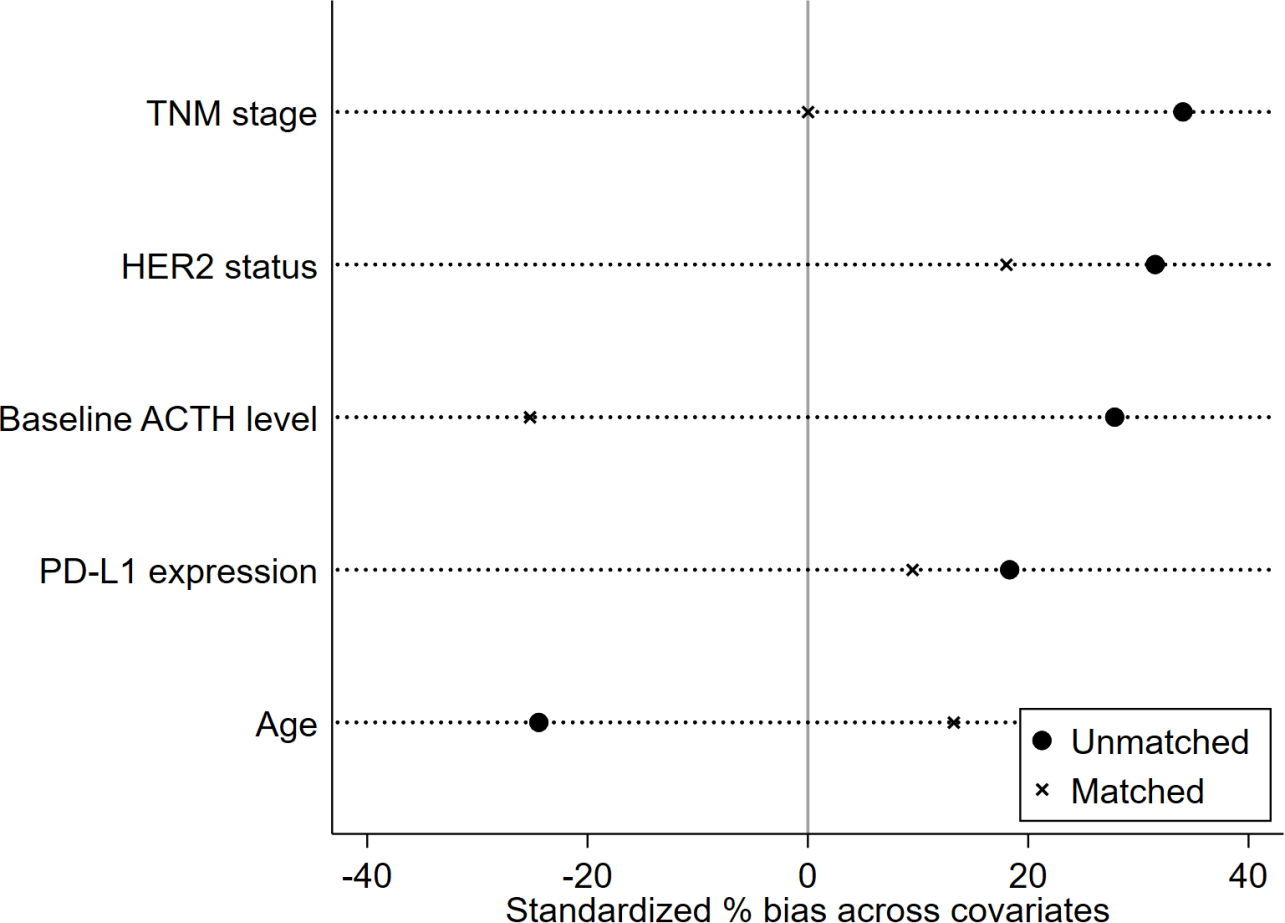
The scatter plot of standardized mean differences.

**Figure 8:**
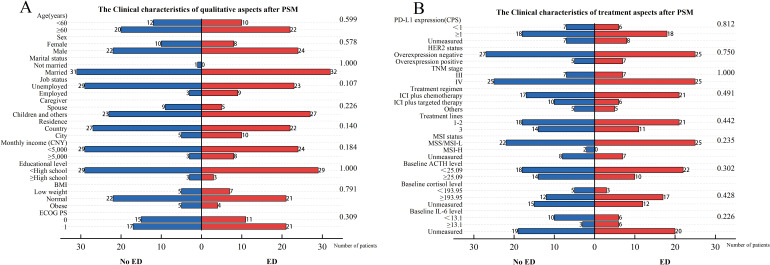
Clinical characteristics for included patients after PSM. The left vertical axis represents the covariates, while the right one corresponds to the P-values obtained from the chi-square test of each covariate against the groups of baseline ED. The horizontal axis reflects the number of patients. Among them, the blue bar graph represents the number of people in the baseline no-ED group, and the red bar graph represents the number of people in the baseline ED group. **(A)** The Clinical characteristics of qualitative aspects after PSM; **(B)** The Clinical characteristics of treatment aspects after PSM.

**Figure 9:**
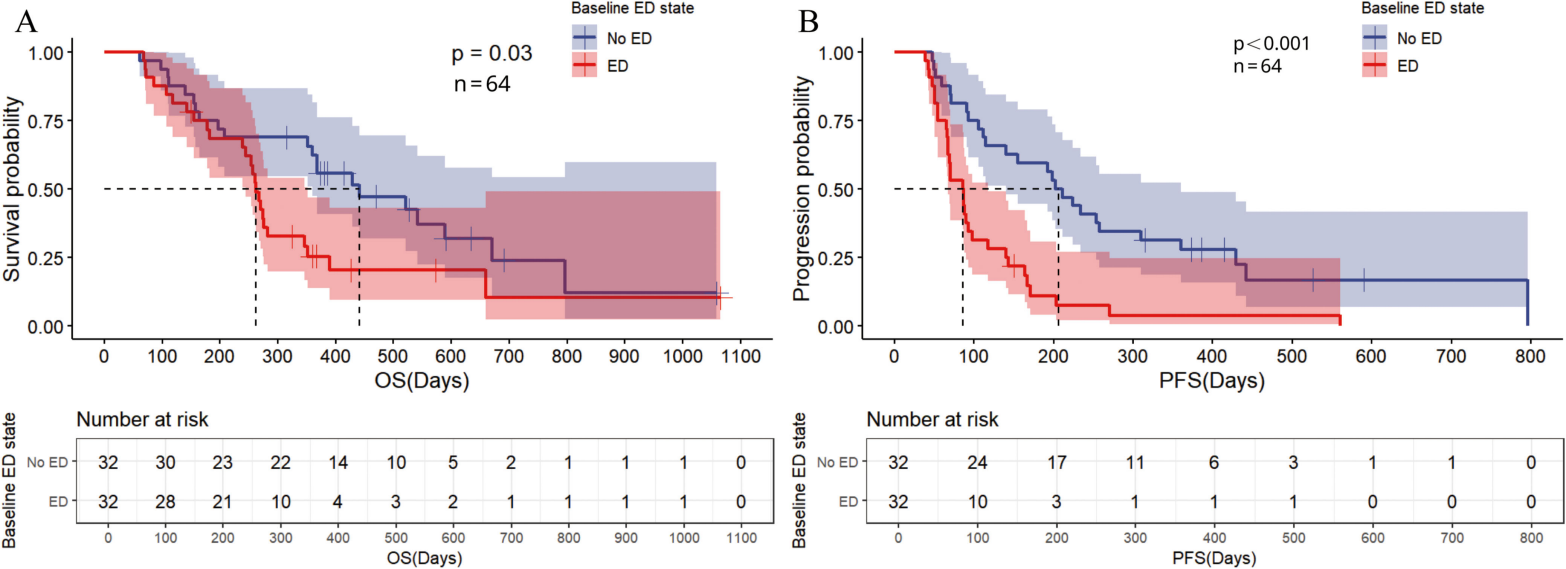
Kaplan Meier curve for baseline ED state after PSM. **(A)** The Kaplan Meier curve for OS; **(B)** The Kaplan Meier curve for PFS.

The propensity score was calculated based on five clinical characteristics, including TNM stage, HER2 status, baseline ACTH level, PD-L1 expression, and age. After matching, there were no significant differences in matched variables between the ED group and no-ED group, indicating that the balance assumption was met. The scatter plot of standardized mean differences for each covariate showed that all matched variables fell within the -0.25 to 0.25 range, which indicated that balance was achieved for all variables (**Figure 7**). The matched data showed that there were 32 cases in the ED group and 32 cases in the no-ED group, with clinical characteristics after PSM shown in **Figures 8A**, **B**. Univariate analysis after PSM still indicated a significant association between ED and shorter PFS(HR:2.35, 95%CI:1.235-4.472, P=0.009), as well as a significant association between ED and shorter OS(HR:2.186, 95%CI:1.043-4.582, P=0.038) following ICIs treatment. Kaplan-Meier curves demonstrated significant intergroup differences in both OS(P=0.034, **Figure 9A**) and PFS (P=0.007, **Figure 9B**) based on ED grouping.

### Survival analysis for dynamic changes of ED status during the ICI treatment

After 2 cycles of ICIs treatment, 80 (76.9%) patients underwent a second assessment of their depression and anxiety states. In the baseline No-ED group, 34 (42.5%) patients were in the ‘never ED’ group and 14 (17.5%) patients were in the ‘new onset ED’ group. In the baseline ED group, 28 (35.0%) patients were in the ‘persistent ED’ group and 12 (15.0%) patients were in the ‘remission from ED’ group.

In the ‘new onset ED’ group of baseline No-ED group, the median OS was 12.133 months (95%CI: 11.093-13.173), while in the ‘never ED’ group of baseline No-ED group, the median OS was 22.333 months (95%CI: 11.108-33.558). There was a significant difference between the two groups with a HR of 2.813 (95%CI:1.270-6.228, P=0.011), suggesting that ‘new onset ED’ was associated with poor survival outcomes of AGC patients undergoing ICIs treatment. In the ‘new onset ED’ group, the median PFS was 3.733 months (95%CI: 3.121-4.345), while in the ‘never ED’ group, the median PFS was 11.333 months (95%CI: 6.490-16.176). There was a significant difference between the two groups with a HR of 3.204 (95%CI: 1.526-6.730, P=0.002), suggesting that ‘new onset ED’ was associated with a higher risk of progression in AGC patients who receive ICIs treatment. The ‘ED persistently’ group from baseline ED group exhibited no significant difference in OS compared to the ‘ED remission’ group from baseline ED group (HR: 1.723, 95% CI: 0.764-3.884, P=0.190), and PFS also showed no significant difference between the two groups (HR: 1.314, 95% CI: 0.634-2.720, P=0.463). This finding indicates that the prognosis for AGC patients who experience alleviation of ED, despite no substantial improvement in overall emotional well-being following ICIs treatment, tends to be more favorable than that of patients with ongoing ED. The Kaplan–Meier curve was showed in **Supplementary Figure S3A**, **S3B**. This indicates that for GC patients with ED, timely implementation of intervention measures may help improve their prognosis during treatment with ICIs.

### Correlation of ED with cortisol and ACTH

Among the 104 patients included in this study, 59 (56.7%) had cortisol levels measured at baseline with 48 (81.3%) in the higher cortisol group and 11 (18.6%) in the lower cortisol group. The missing data rate for cortisol level was 43.3%. In the baseline ED group, 26 (52.2%) patients exhibited higher cortisol levels, compared to 24 patients (41.4%) in the baseline no-ED group. Although a higher proportion of patients in the baseline ED group presented with higher cortisol levels, the difference between the two cohorts was not statistically significant (P = 0.137, **Figure 2B**). Among patients with baseline depression, 21 (91.3%) exhibited higher cortisol levels, whereas among those without baseline depression, 27 (75.0%) demonstrated higher cortisol levels. No statistically significant difference was observed between the two groups (P = 0.174).

The median OS for the higher cortisol group was 13.900 months (95% CI: 10.238-17.562), whereas the median OS for the lower cortisol group has not yet been reached. For patients with AGC who receive ICIs treatment, higher baseline cortisol levels tend to have worse survival outcomes, but the difference is not statistically significant (HR: 2.318, 95% CI: 0.805-6.679, P=0.119, **Figure 3A**).

## Discussion

Numerous studies have demonstrated that anxiety and depression influence the biological behavior of cancer, facilitating cancer development, metastasis, and invasion, which may ultimately result in unfavorable outcomes for patients with cancer (23, 29, 38). Patients with GC are at an increased risk of malnutrition, exhibit a lower BMI and reduced physical activity levels, all of which can adversely impact their quality of life and contribute to ED (26). ED is frequently underestimated in the context of diagnosing and treating GC. ICIs are pivotal in the systemic management of GC, but few studies report the correlation between ED and outcomes in AGC patients receiving ICIs.

Our investigation reveals that baseline ED correlates with poorer survival outcomes, an increased risk of disease progression, and lower rates of tumor response in patients with AGC undergoing ICIs therapy. In the survival analysis after PSM, our results further confirm that ED is independently associated with survival outcomes in patients who receive ICI treatment. This is consistent with the findings of in the NSCLC cohort that ED was associated with a poorer prognosis in patients treated with ICIs (22). Our observations indicate that patients exhibiting baseline depressive symptoms experienced a more pronounced negative impact on the prognosis of AGC patients treated with ICIs, while patients with baseline anxiety were inclined to have poorer survival, albeit without reaching statistical significance. The emergence of new ED during treatment also has a negative predictive effect on the prognosis of GC patients who treated with ICIs.

To date, there have been limited experimental investigations elucidating the specific mechanisms through which ED influences sensitivity and resistance to ICIs. However, it seems that anxiety and depression may modulate the anti-tumor efficacy of ICIs therapy through multiple pathways. In patients with anxiety or depression, the HPA axis is overactivated, leading to the secretion of various stress-related neurotransmitters including catecholamines and cortisol (29). The catecholamines can activate β2-adrenergic receptor-adrenergic receptor (β2-AR) so as to activate the epithelial–mesenchymal transition (EMT) of GC cells via the Janus kinase-signal transducer and activator of transcription (JAK-STAT) pathway (39). The EMT of cancer cells are less susceptible to attack by CD8+ T cells or natural killer cells (NK cells), leading to low response to anti-PD-1/PD-L1 therapy (40). β-receptor blockers can reverse the role of catecholamines in promoting cancer invasion (41), but whether they can reduce the resistance to ICIs needs further basic experimental verification.

Although obesity is a common comorbidity of depression, and the amount of adipose tissue has been demonstrated to be part of the relationship between depression and elevated inflammatory markers (30), our study did not find a significant correlation between BMI and ED. This might be due to the fact that patients with AGC often present with significant weight loss when seeking medical treatment.

Our study with a small sample size did not observe a significant correlation between cortisol level elevation and ED in cancer patients, nor did it observe a statistically significant correlation between cortisol level and the efficacy of ICIs treatment. As for the reasons, apart from the limitation of small sample size, due to the practical difficulties in clinical operation, we were unable to measure the peak levels and rhythms of cortisol and ACTH. However, both of these arguments have been validated in other tumor types studies with larger sample sizes (18, 22). The preceding paragraph mentioned that anxiety and depression can stimulate cortisol levels by over-activating the HPA axis, and real-world studies have also found that patients with tumors and ED have higher serum cortisol levels than those without ED (31). Multiple research found that cortisol can induce functional impairment in CD8+ tumor-infiltrating T lymphocytes (TILs), which may lead to poor responses to ICIs (42). A real-world study further demonstrated that higher endogenous cortisol levels impact the response of advanced cancer patients to ICIs therapy (18). There is also a reports that targeting the glucocorticoid receptor -CCR8 axis in mice significantly suppressed tumor growth (43). The association for glucocorticoid with ED and its impact on ICI efficiency in AGC patients needed to be reevaluated in larger sample size.

Previous studies have found that depression can induce the activation of the enzyme indoleamine 2,3-dioxygenase (IDO) signaling pathway in cancer patients, and IDO can promote the breakdown of tryptophan (44). The degradation products of tryptophan has been proven to inhibit the proliferation of CD8+ T cells and NK cells thereby potentially affecting the effects of ICIs (44). The IDO Pathway was also found to be a factor that mediates the development of resistance to PD-1/PD-L1 inhibitors in phase II clinical trials (44). Patients with depressive symptoms in GC have increased levels of reactive oxygen species in their bodies, which can activate the phosphatidylinositol 3−kinase (PI3K)/protein kinase B (AKT)/mechanistic target of rapamycin (mTOR) signaling pathway to suppress the maturation of dendritic cells(DC), reduce (TILs), and promote the proliferation of immunosuppressive myeloid-derived suppressor cells, thereby reducing the effectiveness of ICIs (45–47). Studies have revealed that the levels of biomarkers that can inhibit the expression of vascular endothelial growth factor (VEGF) in the body, such as dopamine and γ-aminobutyric acid, decrease in patients with ED. This indicates that the ED state may lead to an increase in VEGF expression, thereby affecting the therapeutic efficacy of ICIs (48). Numerous studies have found that high levels of serum IL-6 and IL-8 in cancer patients are significantly associated with anxiety and depressive states, and IL-6 and IL-8 have also been validated clinically to be associated with poorer responses to ICIs treatment (13, 23). We will measure the levels of IL-6 and IL-8 to evaluate their influence for both ICI efficiency and ED occurrence in the future cohorts. The above multiple mechanisms to some extent explain how ED, especially depression, can mediate poor responses and resistance to ICIs.

The guidelines of the American Society of Clinical Oncology also emphasize the importance of regular assessment of depression and anxiety in cancer patients (49). Unfortunately, our study did not observe a significant impact of ED relief on survival outcomes due to the limitation of sample size. However, the NSCLS patients who experienced ED relief exhibited better survival outcomes compared to those with persistent ED (22). The mindfulness training cognitive-behavioral therapy and music therapy were recommended to improve psychological stress during cancer treatment (23, 50). For GC patients experiencing ED, psychological intervention, from a macroscopic perspective, improves both the quality of life and treatment adherence. From a microscopic perspective, by modulating the TME that fosters cancer progression, it may partially improve resistance to ICIs therapy (51). Research has demonstrated that psychological intervention combined with antidepressant medications reduces pro-inflammatory cytokines such as TNF-α, IL-6, and IFN-γ, while also improving HPA axis responsiveness, which could potentially synergize with ICIs therapy (30, 52).

Our study also has other inevitable limitations. Firstly, this research is an observational study with a small sample size, which inherently introduces a selection bias that cannot be avoided and restricts the demonstration efficacy of this study. Secondly, we evaluated the symptoms of depression and anxiety in patients using questionnaires, which are inherently subjective and may introduce information bias. Thirdly, while we endeavored to minimize confounding factors through COX multivariate analysis and PSM, the treatment regimens, ICIs drug choices, and number of treatment lines used by the included patients were not consistent, so it was impossible to entirely eliminate confounding influences. Our study did not evaluate the impact of factors such as opioid use, socioeconomic support, and comorbidities on ED status and survival. We also did not explore factors that are difficult to quantify and evaluate, such as the surrounding environment, personal past mental health status, and social support. We will performed further study to check whether they would interfere with the relationship between ED and prognosis. Finally, In terms of exploring the biomarkers of the impact of ED on ICIs treatment, this study only conducted detections on cortisol and ACTH. However, for other potentially relevant biomarkers, such as cortisol rhythm, ACTH rhythm, catecholamines, cytokines (IL-6, IL-8), and immune cells (CD8^+^ T), etc., in-depth exploration is still awaited. In light of these limitations, it is necessary to undertake prospective, multicenter clinical studies with large sample sizes for validation.

## Conclusion

Baseline ED is a negative predictor of outcomes for patients with AGC receiving ICIs treatment, with depression having a greater impact on outcomes. ED experienced during treatment may also influence survival outcomes. Regularly monitoring the anxiety and depression state of patients with AGC during ICIs therapy, coupled with timely interventions, may enhance their overall prognosis.

## Data Availability

The original contributions presented in the study are included in the article/**Supplementary Material**. Further inquiries can be directed to the corresponding author.

## Glossary

ED: Emotional distress
TME: Tumor microenvironments
IARC: International Agency for Research on Cancer
GC: Gastric cancer
AGC: Advanced gastric cancer
ICIs: Immune checkpoint inhibitors
OS: Overall survival
PFS: Progression-free survival
ORR: Objective response rate
DCR: Disease control rate
CR: Complete response
PR: Partial response
SD: Stable disease
TLS: Tertiary lymphoid structures
DC: Dendritic cells
CRP: C-reactive protein
RR: Risk ratio
HR: Hazard ratio
HPA: Hypothalamic-pituitary-adrenal
NSCLC: Non-small-cell lung cancer
PD-1: Programmed cell death protein 1
PD-L1: Programmed cell death-ligand 1
AJCC: American Joint Committee on Cancer
CT: Computed tomography
ECOG PS: Eastern Cooperative Oncology Group performance status
RECIST v1.1: Version 1.1 of the Response Evaluation Criteria in Solid Tumors
PHQ-9: 9-item Patient Health Questionnaire
GAD-7: 7-item Generalized Anxiety Disorder Questionnaire
ACTH: Adrenocorticotropic hormone
ROC: Receiver operating characteristic
CPS: Combined positive score
HER2: Human epidermal growth factor receptor 2
TNM: Tumor-Node-Metastasis
MSI: Microsatellite instability
CI: Confidence interval
PSM: Propensity score matching
PD-1: Programmed cell death protein 1
β2-AR: β2-adrenergic receptor-adrenergic receptor
EMT: Epithelial–mesenchymal transition
JAK-STAT: Janus kinase-signal transducer and activator of transcription
NK cells: Natural killer cells
TILs: Tumor-infiltrating T lymphocytes
IDO: Indoleamine 2,3-dioxygenase
PI3K: Phosphatidylinositol 3-kinase
AKT: protein kinase B
mTOR: Mechanistic target of rapamycin
VEGF: Vascular endothelial growth factor
DC: Dendritic cells
BMI: Body Mass Index
