# Supplementary material for: Association between emotional distress and the efficacy of advanced gastric cancer patients undergoing treatment with immune checkpoint inhibitors: a cohort study and propensity score matching study
Source: Front Oncol. 2025 Jul 25;15:1516643. doi: 10.3389/fonc.2025.1516643 (PMC12331473; doi:10.3389/fonc.2025.1516643)
Supplement: Supplementary file 1 [file DataSheet1.docx]

**Supplementary figures and tables：**

**Table.S1.** P-values from univariate analyses of OS for each Covariate were corrected using the Benjamini-Hochberg method.

| Covariate | P | Benjamini-Hochberg critical value********* | Conclusion based on 10% FDR |
| --- | --- | --- | --- |
| Depressive state | <0.001 | 0.00625 | Significant |
| Baseline ED state | 0.003 | 0.0125 | Significant |
| never ED vs.ED new onset | 0.008 | 0.01875 | Significant |
| TNM stage | 0.012 | 0.025 | Significant |
| Treatment lines | 0.043 | 0.03125 | False positive |
| ECOG PS | 0.045 | 0.0375 | False positive |
| PD-L1 expression（CPS） | 0.118 | 0.04375 | Not Significant |
| Baseline cortisol level | 0.119 | 0.05 | Not Significant |
| Sex | 0.155 | 0.05625 | Not Significant |
| ED remission vs.ED persistently | 0.19 | 0.0625 | Not Significant |
| Treatment regimen | 0.302 | 0.06875 | Not Significant |
| Baseline ACTH level | 0.355 | 0.075 | Not Significant |
| Aanxiety state | 0.383 | 0.08125 | Not Significant |
| Age | 0.413 | 0.0875 | Not Significant |
| MSI status | 0.617 | 0.09375 | Not Significant |
| HER2 status | 0.943 | 0.1 | Not Significant |

*****Benjamini-Hochberg critical value=(k/M)*α, k denotes the rank of a p-value after all p-values are sorted in ascending order**;** M is the total number of P-values**;** *α* is the FDR threshold, which is set to 0.1.

**
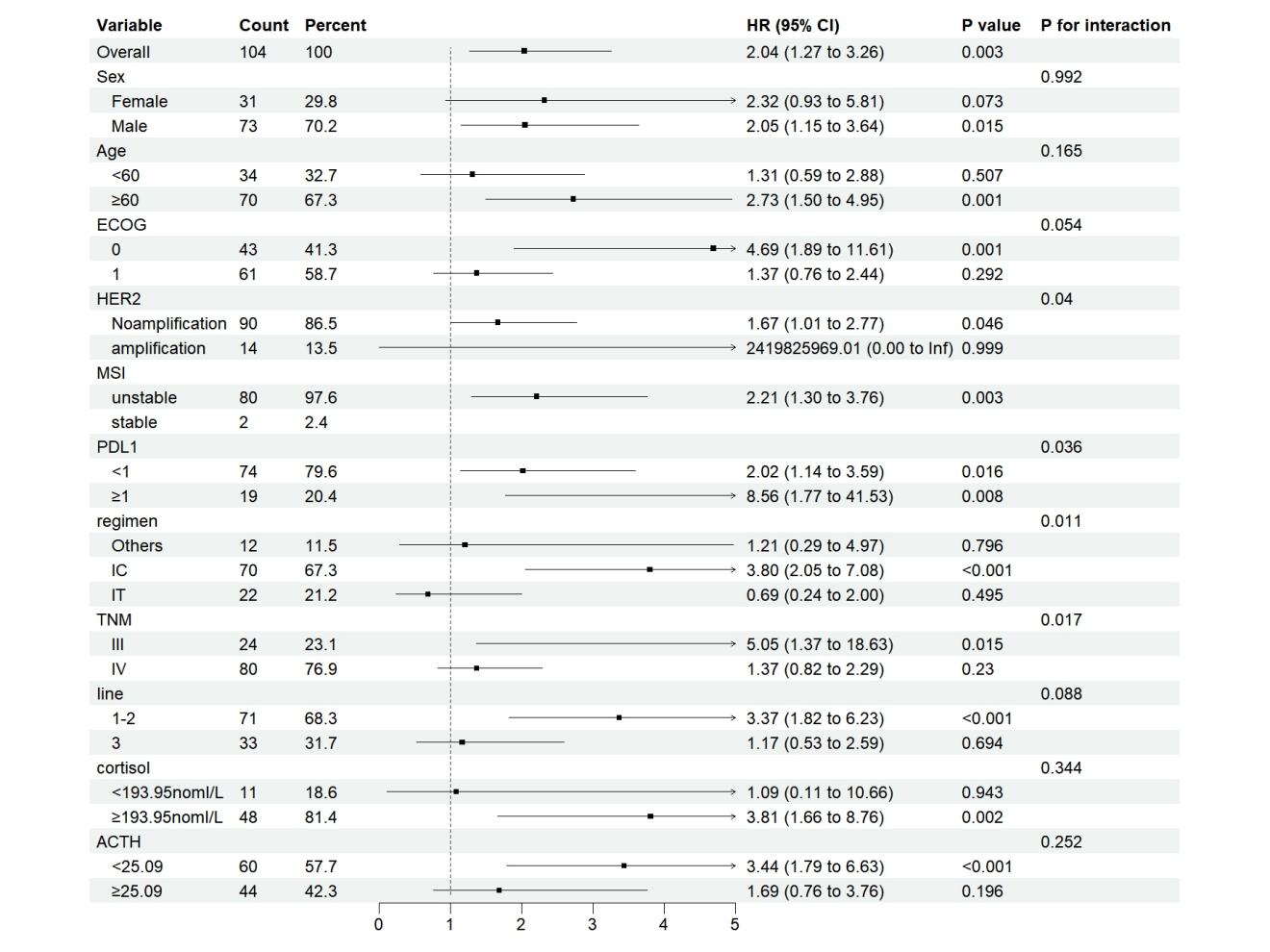
Figure.S1.** The subgroup analysis of OS according to baseline ED state.

**Table.S2.** P-values fromsubgroup analyses of OS according to baseline ED state were corrected using the Benjamini-Hochberg method.

| P(k) | P | Benjamini-Hochberg critical value | Conclusion based on 10% FDR |
| --- | --- | --- | --- |
| P(1) | ﹤0.001 | 0.004347826 | Significant |
| P(2) | ﹤0.001 | 0.008695652 | Significant |
| P(3) | ﹤0.001 | 0.013043478 | Significant |
| P(4) | ﹤0.001 | 0.017391304 | Significant |
| P(5) | ﹤0.001 | 0.02173913 | Significant |
| P(6) | 0.002 | 0.026086957 | Significant |
| P(7) | 0.003 | 0.030434783 | Significant |
| P(8) | 0.003 | 0.034782609 | Significant |
| P(9) | 0.008 | 0.039130435 | Significant |
| P(10) | 0.015 | 0.043478261 | Significant |
| P(11) | 0.015 | 0.047826087 | Significant |
| P(12) | 0.016 | 0.052173913 | Significant |
| P(13) | 0.046 | 0.056521739 | Significant |
| P(14) | 0.073 | 0.060869565 | False positive |
| P(15) | 0.196 | 0.065217391 | Not Significant |
| P(16) | 0.230 | 0.069565217 | Not Significant |
| P(17) | 0.292 | 0.073913043 | Not Significant |
| P(18) | 0.495 | 0.07826087 | Not Significant |
| P(19) | 0.507 | 0.082608696 | Not Significant |
| P(20) | 0.694 | 0.086956522 | Not Significant |
| P(21) | 0.796 | 0.091304348 | Not Significant |
| P(22) | 0.943 | 0.095652174 | Not Significant |
| P(23) | 0.999 | 0.1 | Not Significant |

**Table.S3.** P-values from univariate analyses of PFS for each Covariate were corrected using the Benjamini-Hochberg method.

| Covariate | P | Benjamini-Hochberg critical value | Conclusion based on 10% FDR |
| --- | --- | --- | --- |
| Baseline ED state | ﹤0.001 | 0.00625 | Significant |
| Depressive state | ﹤0.001 | 0.0125 | Significant |
| Treatment lines | ﹤0.001 | 0.01875 | Significant |
| never ED vs.ED new onset | 0.002 | 0.025 | Significant |
| Aanxiety state | 0.06 | 0.03125 | Not Significant |
| ECOG PS | 0.066 | 0.0375 | Not Significant |
| Baseline ACTH level | 0.08 | 0.04375 | Not Significant |
| PD-L1 expression（CPS） | 0.139 | 0.05 | Not Significant |
| Age | 0.17 | 0.05625 | Not Significant |
| TNM stage | 0.201 | 0.0625 | Not Significant |
| Treatment regimen | 0.264 | 0.06875 | Not Significant |
| ED remission vs.ED persistently | 0.463 | 0.075 | Not Significant |
| MSI status | 0.474 | 0.08125 | Not Significant |
| Sex | 0.669 | 0.0875 | Not Significant |
| Baseline cortisol level | 0.867 | 0.09375 | Not Significant |
| HER2 status | 0.902 | 0.1 | Not Significant |

**
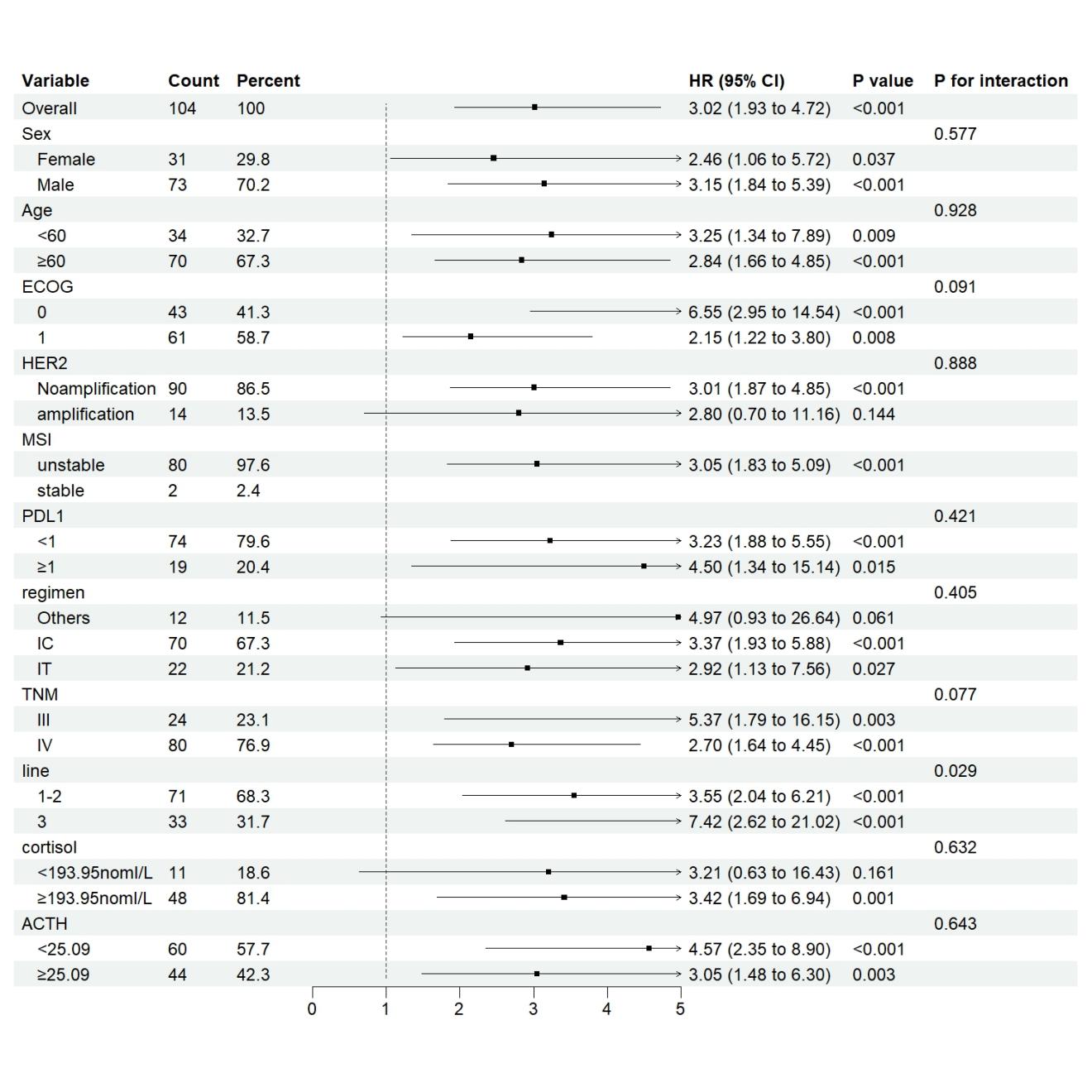
Figure.S2.** The subgroup analysis of PFS according to baseline ED state.

**Table.S4.** P-values fromsubgroup analyses of OS according to baseline ED state were corrected using the Benjamini-Hochberg method.

| P(k) | P | Benjamini-Hochberg critical value | Conclusion based on 10% FDR |
| --- | --- | --- | --- |
| P(1) | ﹤0.001 | 0.004347826 | Significant |
| P(2) | ﹤0.001 | 0.008695652 | Significant |
| P(3) | ﹤0.001 | 0.013043478 | Significant |
| P(4) | ﹤0.001 | 0.017391304 | Significant |
| P(5) | ﹤0.001 | 0.02173913 | Significant |
| P(6) | ﹤0.001 | 0.026086957 | Significant |
| P(7) | ﹤0.001 | 0.030434783 | Significant |
| P(8) | ﹤0.001 | 0.034782609 | Significant |
| P(9) | ﹤0.001 | 0.039130435 | Significant |
| P(10) | ﹤0.001 | 0.043478261 | Significant |
| P(11) | ﹤0.001 | 0.047826087 | Significant |
| P(12) | ﹤0.001 | 0.052173913 | Significant |
| P(13) | 0.001 | 0.056521739 | Significant |
| P(14) | 0.003 | 0.060869565 | Significant |
| P(15) | 0.003 | 0.065217391 | Significant |
| P(16) | 0.008 | 0.069565217 | Significant |
| P(17) | 0.009 | 0.073913043 | Significant |
| P(18) | 0.015 | 0.07826087 | Significant |
| P(19) | 0.027 | 0.082608696 | Significant |
| P(20) | 0.037 | 0.086956522 | Significant |
| P(21) | 0.061 | 0.091304348 | Significant |
| P(22) | 0.144 | 0.095652174 | Not Significant |
| P(23) | 0.161 | 0.1 | Not Significant |


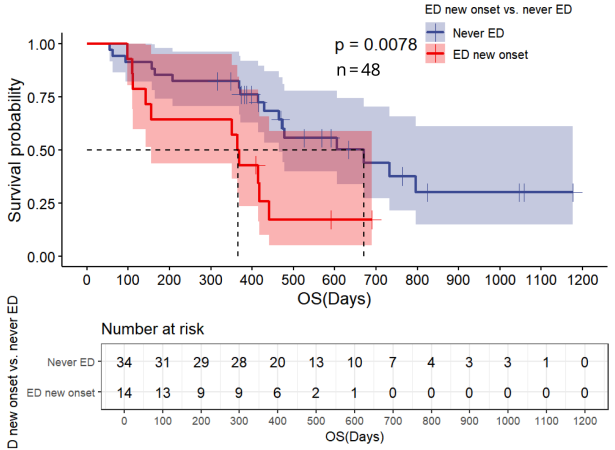


A


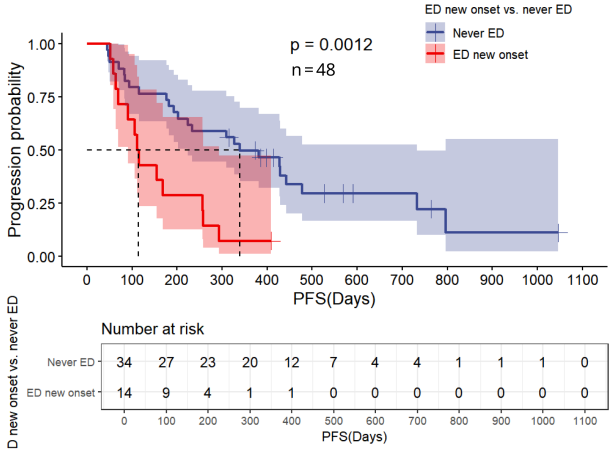


B

**Figure.S3.** Kaplan Meier curve for OS and PFS for ED new onset vs. never ED. A: OS for ED new onset vs. never ED; B: PFS for ED new onset vs. never ED.
